# Jugulotympanic Glomus: A Case Report on a Rare Presentation of a Vascular Middle Ear Tumor

**DOI:** 10.7759/cureus.90311

**Published:** 2025-08-17

**Authors:** Maria J Heredia, Katia Y Lopez-Zamarron, Eliany Leon Figueredo, Elena Kate Ligori Vasquez, Daniela Enriquez Chavez, Mayra Isabel Jarquin Cervantes

**Affiliations:** 1 Cardiology, Ministry of Public Health of Ecuador, Quito, ECU; 2 Health Sciences Division, University of Monterrey, San Pedro Garza Garcia, MEX; 3 General Medicine, Englewood Health Physician Network - Primary Care, New Jersey, USA; 4 School of Medicine, Faculty of Health Sciences, Peruvian University of Applied Sciences, Lima, PER; 5 Faculty of Human Medicine, Universidad de San Martin de Porres, Lima, PER; 6 School of Medicine, University of the Sierra Sur, Miahuatlán de Porfirio Díaz, MEX

**Keywords:** bezold abscess, glomus tumor, jugulotympanic paraganglioma, mastoiditis, otitis media complications

## Abstract

Jugulotympanic paragangliomas (JTPs) are neuroendocrine tumors, frequently benign, that originate from paraganglia cells. Due to their small initial size and slow growth, they tend to be asymptomatic for long periods, leading to delayed presentation. Our case report describes a JTP complicated by mastoiditis and a Bezold abscess. We report the case of a 70-year-old female with a history of hypertension and an ischemic stroke 20 years prior, who was diagnosed with an unresectable JTP via MRI. At the time of presentation, she reported progressive ear pain, fever, and a tender mass behind the ear that had developed over the preceding two weeks. On examination, findings were consistent with mastoiditis, and otoscopy of the right ear showed malodorous otorrhea partially obscuring the external canal, a perforated tympanic membrane, and a red-violaceous, pulsatile mass behind it, for which, along with the already diagnosed JTP, she was admitted to the hospital. The patient experienced recurrent infections not typically associated with this tumor type. Well-known complications are otalgia, hoarseness of voice, and nerve palsy involving the facial nerve (cranial nerve VII) -- deficits associated with the mass effect near nerves found adjacent to the neck and ear. It is rare to discover mastoiditis or a Bezold abscess in this population, with only a few cases being reported worldwide. Considering the paraganglioma remained at its initial size, this did not correlate with the complications; instead, the obstruction of the ear canal, negative pressure, and tubal dysfunction created favorable conditions for bacterial growth, predisposing to infections. Strict follow-up is essential for each patient to enable early diagnosis at a treatable stage, thereby reducing adverse outcomes.

Given the limited published data on this rare occurrence, we aim to contribute to the existing case reports and highlight the need for further research on uncommon paragangliomas to optimize management strategies and anticipate potential complications.

## Introduction

Paraganglia are neuroendocrine cells derived from the neural crest and distributed along both the sympathetic and parasympathetic chains. In the ear, they are called jugulotympanic paraganglia [1]. Tumors arising from these cells are called paragangliomas. Those originating from sympathetic paraganglia can increase catecholamines, while those from parasympathetic ganglia are generally non-functioning. Parasympathetic paragangliomas are rare, with an estimated incidence of 0.07 cases per 100,000 people per year [2]. Most patients are diagnosed between the third and fifth decades of life, with an average age of 47 years, and the majority are female [3]. In general, paragangliomas are benign tumors; for instance, in the United States, only 93 cases per 400 million reported in 2002 were classified as malignant [4].

Clinical presentation varies from asymptomatic cases to those with severe complications, making early evaluation essential. Although paragangliomas are uncommon, a few have been reported in association with mastoiditis or a Bezold abscess, both of which are rare conditions themselves. Notably, only 0.002% of children with otitis media develop mastoiditis, and merely 100 cases of Bezold's abscesses have been documented so far [5,6].

We present a case of an unresectable stage IV jugulotympanic glomus of 20 years of evolution that developed unusual complications. Despite its advanced stage, the patient responded favorably to drainage and antibiotic therapy.

## Case presentation

A 70-year-old woman presented to the emergency room with otalgia, neck pain, and fever. The patient had a history of an ischemic stroke 20 years prior, attributed to long-standing hypertension, and a jugulotympanic glomus tumor was also revealed during that period. Because the tumor was diagnosed at an unresectable stage, her medical team decided on observational management and only addressed coexistent infections with local antibiotics.

The patient began two weeks ago with otalgia; she had experienced similar episodes of acute otitis media in the past that were treated successfully. Seven days after symptom onset, she was evaluated by an otolaryngologist (ENT) and was prescribed clindamycin and amoxicillin with clavulanic acid, but her symptoms remained and progressed, extending to the neck and limiting her mobility; therefore, she had to be taken to the emergency room. In addition, she reported fever and the presence of a tender mass behind the ear.

**Figure 1:**
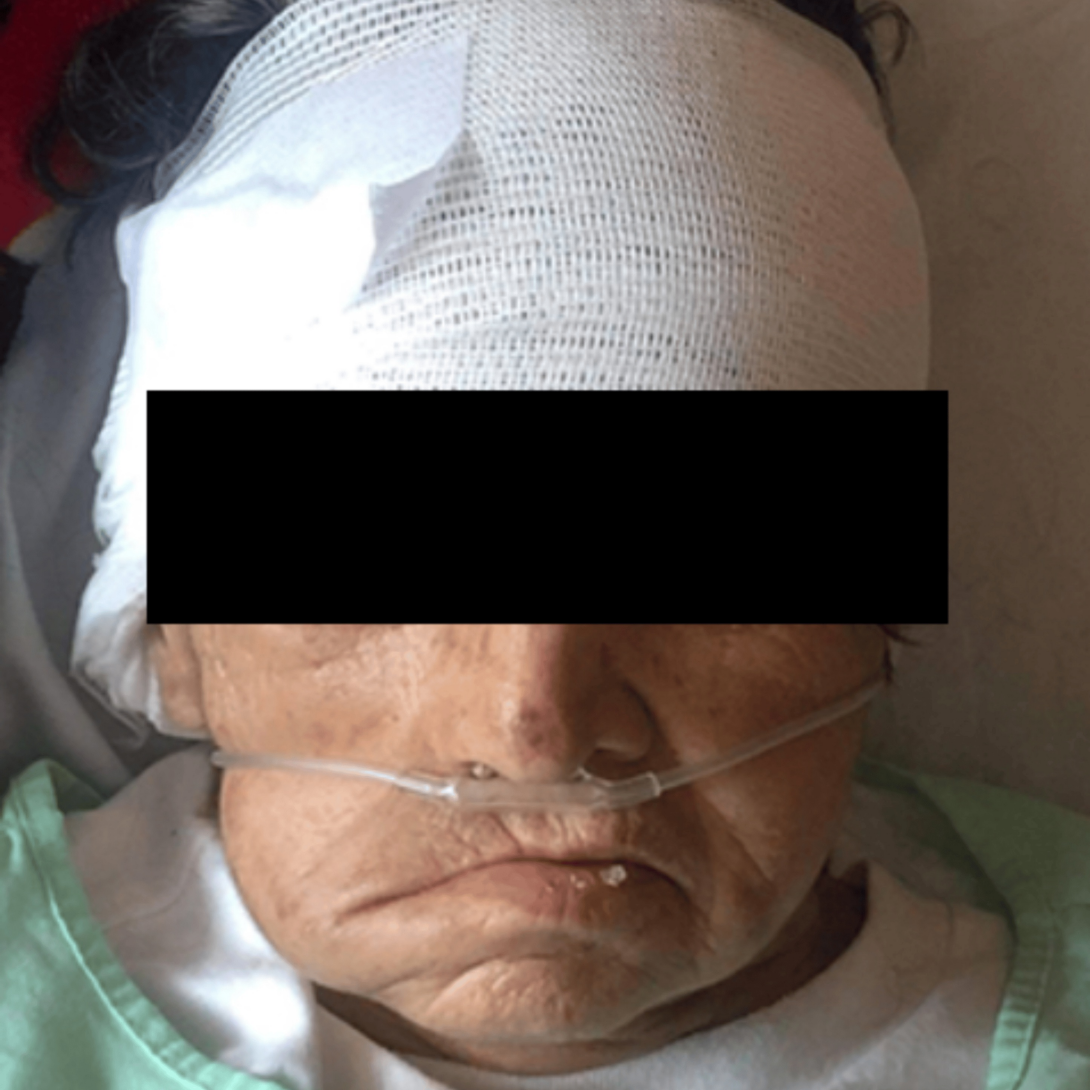
Presence of facial paralysis with deviation of the lip commissure

During her assessment, vital signs were unremarkable, and a neurological exam revealed right-sided facial paralysis (Figure 1).

Examination of the right ear evidenced swelling and tenderness of the mastoid area that extended behind the occipital and parietal region with an erythematous and purplish appearance. Otoscopy of the right ear showed malodorous otorrhea partially obscuring the external canal; it revealed a perforated tympanic membrane and a red-violaceous, pulsatile mass behind it. The left ear examination was unremarkable. These findings led to urgent hospital admission for ENT consultation.

Laboratory exams revealed leukocytosis with neutrophilia and elevated acute phase reactants (Table 1).

**Table 1:** Laboratory exams

Type	Value	Reference range	Unit
CRP	Greater than 150	<6	mg/dL
Procalcitonin	>75	<0.5	ng/mL
Hemoglobin	12.1	12-16	g/dL
Hematocrit	36.2	36-46	%
Mean corpuscular volume	88.2	80-100	fL
Leukocytes	16.92	4.5-11	x103/uL
Lymphocytes	5.7	20-45	%
Neutrophils	91.4	50-70	%
Glucose	127	70-99	mg/dL
Chloride	103	96-106	mEq/L
Sodium	135.8	135-145	mEq/L
Urea	39	10-11	mg/dL
Creatinine	0.49	0.5-1.1	mg/dL

**Figure 2:**
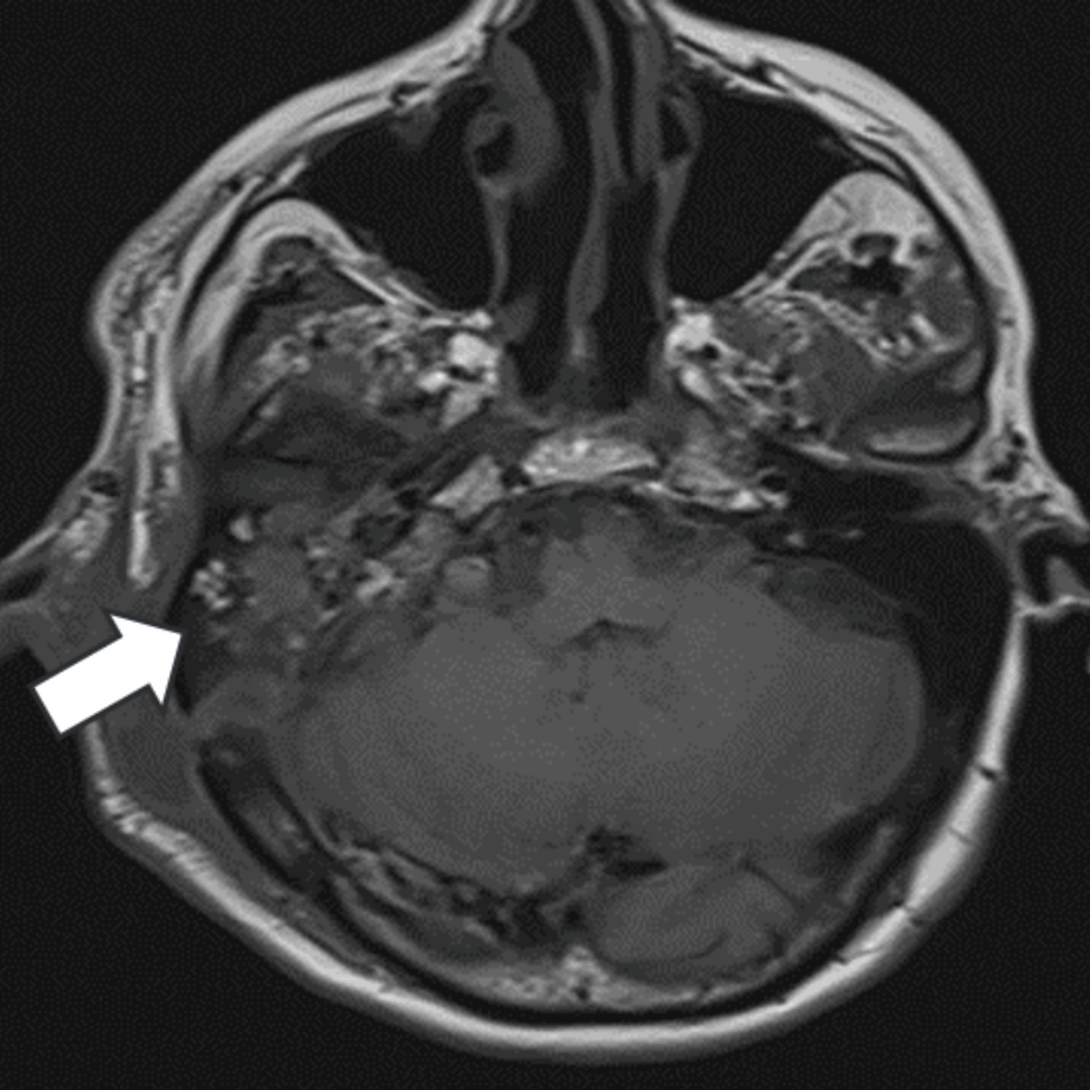
Jugulotympanic glomus Right-sided jugulotympanic glomus (white arrow)

**Figure 3:**
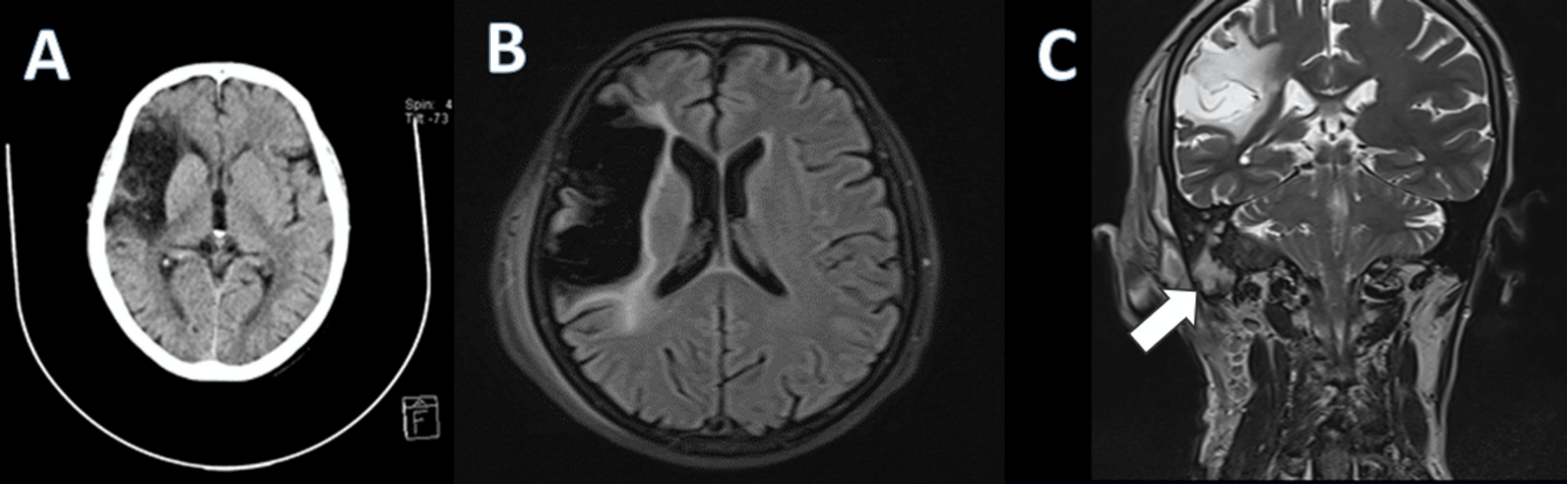
Presence of leukoencephalomalacia on computed tomography and MRI A. Axial CT: Chronic ischemic stroke; B. Axial FLAIR MRI: Chronic ischemic stroke and gliosis; C. Coronal T2 MRI: Jugulotympanic glomus (white arrow) FLAIR: fluid-attenuated inversion recovery

Imaging was obtained, and the MRI showed a right-sided expansive, heterogeneous mass with a characteristic salt-and-pepper appearance, with a small flow void in the internal jugular vein consistent with a grade IV jugulotympanic glomus tumor (Figure 2) that compromises facial (VII) and vestibulocochlear (VIII) nerves. Fluid-attenuated inversion recovery (FLAIR) sequences showed extensive leukoencephalomalacia related to a previous stroke (Figure 3).

**Figure 4:**
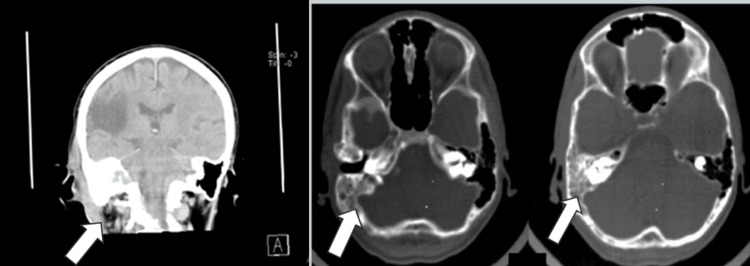
Lack of pneumatization in the right mastoid area with destruction of mastoid air cells (white arrows) showing mastoiditis

**Figure 5:**
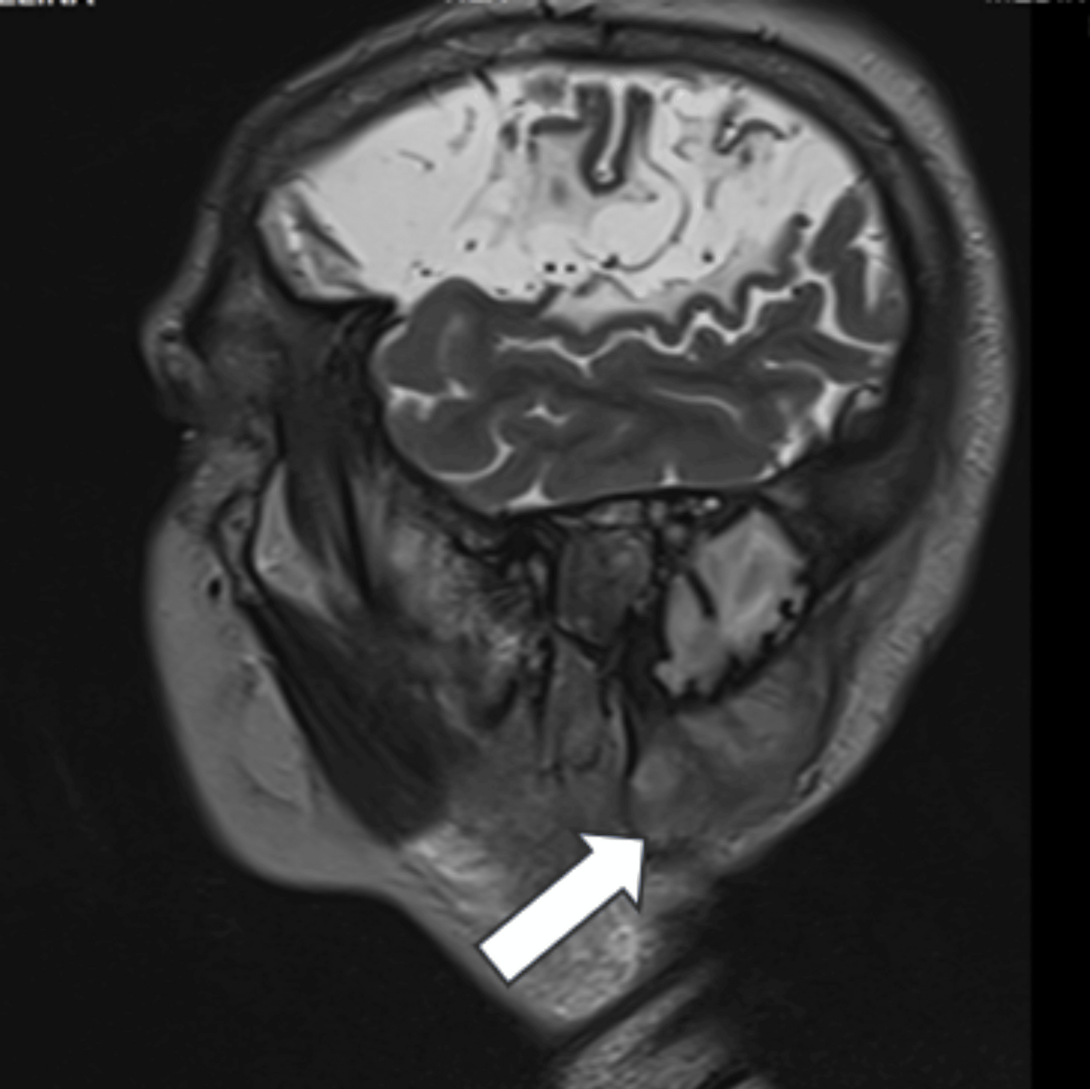
Presence of the Bezold abscess on MRI (white arrow)

In comparison with the previous MRI, no tumor growth was observed. Cranial CT demonstrated right parietotemporal hypodensity and identified a septated soft tissue collection with an air-fluid level in the right hemicranium (Figure 4). In addition, collection and gas are seen in the soft tissues of the mastoid consistent with a Bezold abscess (Figure 5).

**Figure 6:**
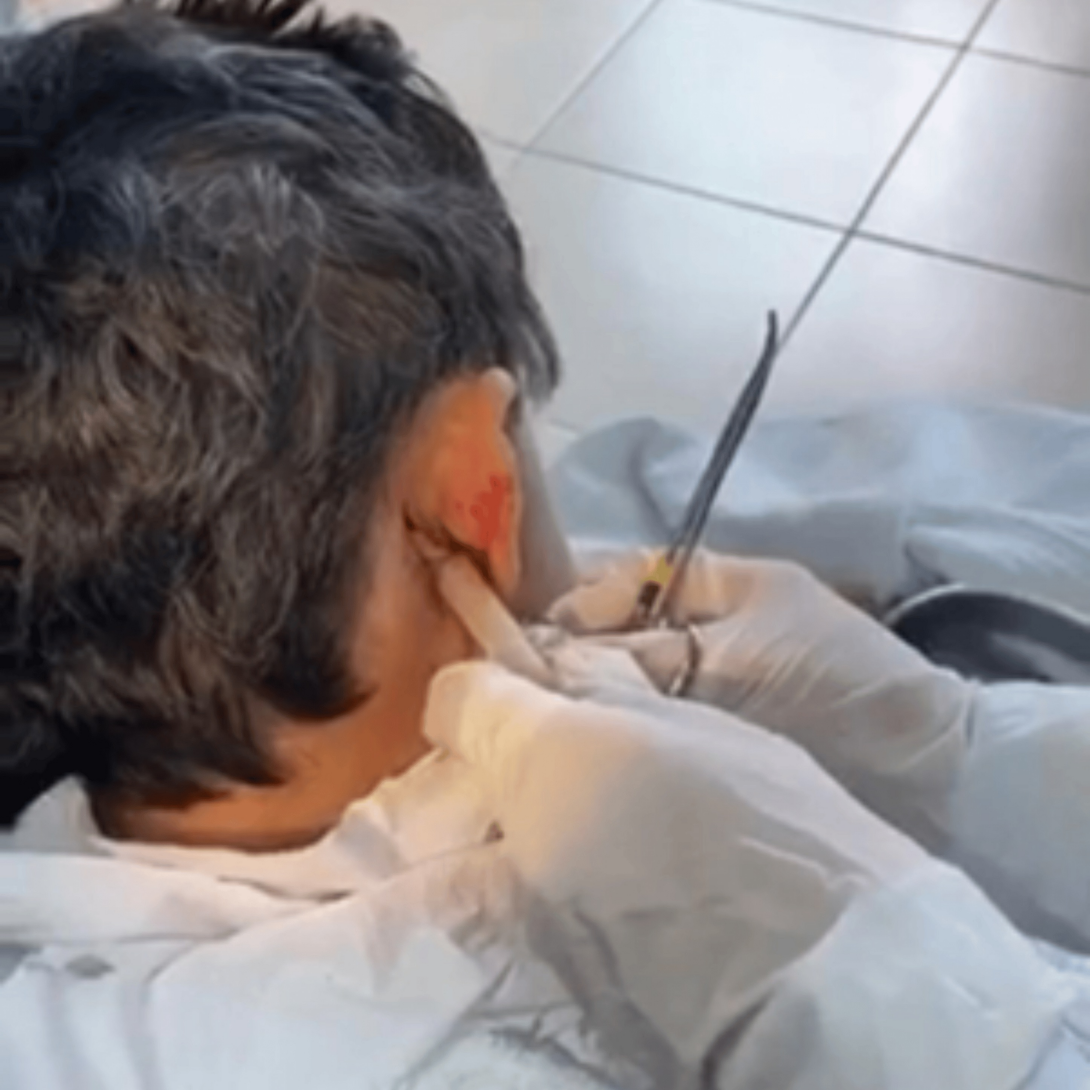
Drainage of Bezold's abscess

**Figure 7:**
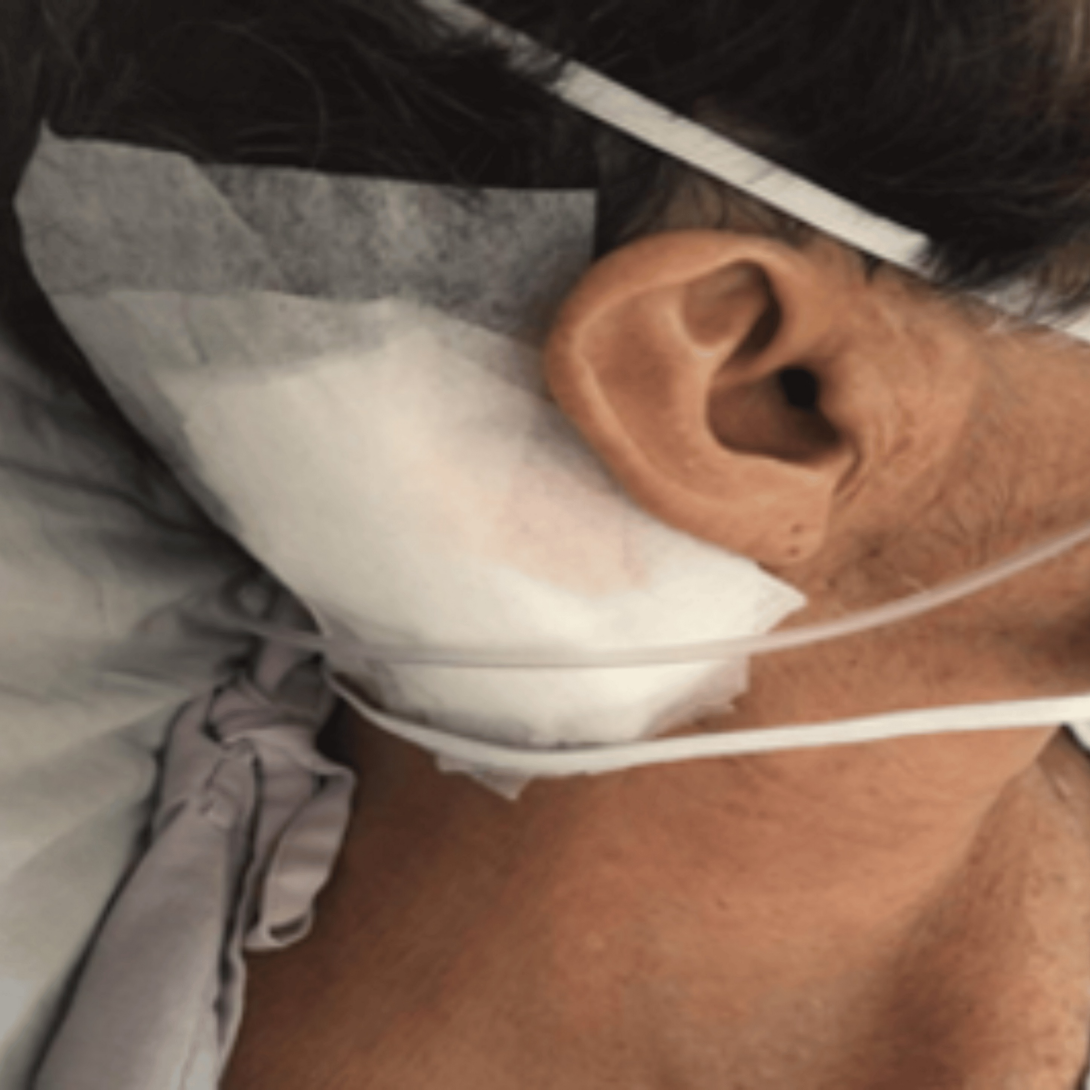
Healing with clean dressings of the drainage area

After admission, it was decided to perform a puncture of the cervical abscess, obtaining 250 ml of purulent material that was sent for culture (Figure 6). In addition, a Penrose drain was inserted. A double antibiotic regimen (ceftriaxone 2 grams IV each day in addition to vancomycin 1 gram every 12 hours) was administered. The patient became afebrile after five days of antibiotic therapy. Mastoid dressing with sodium chloride and hydrogen peroxide was performed to decrease inflammation; the Penrose drain remained functional, draining a small amount of hemopurulent material over the next few days (Figure 7). 

Antibiotic sensitivity test confirmed susceptibility to prescribed antibiotics, and it was decided to continue with the same scheme for an extra three weeks, improving the patient's clinical evolution and allowing the Penrose drain to be removed. After four weeks of hospitalization, the patient was discharged with topical ear drops containing ciprofloxacin 0.3% and dexamethasone 0.1%, oral analgesics, and scheduled outpatient follow-up with ENT.

## Discussion

The patient described in this case was initially diagnosed with a rare parasympathetic paraganglioma that later became complicated with mastoiditis and a Bezold abscess. Jugulotympanic paragangliomas are typically small tumors. Unlike other paragangliomas, these tumors cause symptoms related to mass effect. Some common symptoms are pulsatile tinnitus as the main complaint, sometimes accompanied by conductive hearing loss, otalgia, vertigo, dysphagia, hoarseness, facial nerve palsy, and lower nerve palsy [7]. On examination, a characteristic finding is a pulsating mass behind the tympanic membrane with increased vascularity [8].

When suspected, a diagnosis can be made through a CT to identify bone destruction or an MRI demonstrating a salt-and-pepper appearance, which is characteristic of the disease. Imaging findings can be categorized according to the Fisch or Glasscock/Jackson classification systems [9,10]. Upon confirmation, treatment options vary based on patient and tumor characteristics and may include surgical intervention, observation, or radiotherapy. Overall, radiotherapy is generally preferred due to its lower risk of complications such as bleeding, infection, or significant nerve injury [11].

Although the patient presented with typical symptoms, she also developed a perforated tympanic membrane, malodorous otorrhea, and lab exams consistent with infection, none of which are regular manifestations. Diagnosis was confirmed through CT and MRI, but, in addition, mastoiditis and a Bezold abscess were identified. These findings, combined with the delayed diagnosis of the paraganglioma and its associated tissue destruction, created an environment favorable for pathogen growth, which explains the atypical presentation and recurrence of symptoms.

Considering this unique presentation and the absence of tumor growth, the medical team decided to continue expectant management for the paraganglioma and to treat complications with antibiotics and corticosteroids.

According to a recent systematic review and meta-analysis where the effectiveness of surgery versus radiotherapy was compared, implementation of radiotherapy had higher tumor recurrence (3.5% versus 3.9%) but fewer complication rates (7.6% versus 29.6%), suggesting greater promise with this approach [12]. No data regarding conservative management were found.

In-depth research on the topic discovered that data from paragangliomas was centered on sympathetic paragangliomas, such as pheochromocytoma, and much of the information corresponding to parasympathetic ganglia, including jugulotympanic and vagal paraganglioma, is lacking, probably because of the low incidence. This case was highly unusual since few case reports mentioned complications like mastoiditis and a Bezold abscess. A similar report to our case was found, with the exception that instead of a jugulotympanic paraganglioma, a tympanic paraganglioma was involved. The same antibiotic and debridement therapy was performed, successfully resolving the infection, though recurrence persisted [13].

Another noteworthy aspect of this case is the presence of mastoiditis in this population, since it occurs more frequently in pediatric patients. Nevertheless, it is important to note that the patient has a jugulotympanic paraganglioma that predisposes to repeated infections of otitis media, facilitating mastoiditis and, therefore, a Bezold abscess. This vulnerability arises because the paraganglioma blocks the ear canal, leading to negative pressure and dysfunction of the Eustachian tube. This results in inflammation, promoting the buildup of fluids and secretions, which, in turn, creates an environment conducive to bacterial growth.

We agree that more research on these tumors is needed to confirm superior outcomes using radiotherapy over other therapeutic options, anticipate possible complications, and guide treatment strategies.

## Conclusions

This case illustrates an atypical presentation of an advanced jugulotympanic glomus complicated by mastoiditis and Bezold's abscess, which is not a common presentation due to the current use of antibiotics. As seen in this particular case, the initial symptoms were ear pain and recurrent otitis media, so nothing further was suspected until the perforation of the eardrum and the presence of a painful mass behind the ear were identified.

This case highlights the importance of considering secondary infections in patients with chronic middle ear masses due to jugulotympanic paraganglioma, enabling timely diagnosis and avoiding future complications. The authors recommend that health professionals maintain strict follow-up of these cases to avoid adverse outcomes. Also, due to the low incidence of this pathology, it is essential to promote more research that can evaluate the effectiveness of current treatments, particularly the potential of radiotherapy as a lower-morbidity alternative to surgery.
